# Supplementary material for: The Association between Difficulties with Speech Fluency and Language Skills in a National Age Cohort of Children with Down Syndrome
Source: Brain Sci. 2021 May 26;11(6):704. doi: 10.3390/brainsci11060704 (PMC8226845; doi:10.3390/brainsci11060704)
Supplement: Supplementary file 1 [file brainsci-11-00704-s001.zip › brainsci-1207925-supplementary.pdf]

Table S1. Correlation matrix (N = 41)

	2.	3.	4.	5.	6.	7.	8.	9.	10.
1. Difficulties with fluency	-.20	***-.52***	**-.32**	**-.40**	-.30	-.03	.07	-.21	-.06
2. Nonverbal mental functioning		***.57***	***.60***	**.41**	**.41**	.05	.16	-.02	**.35**
3. Vocabulary, mean			***.70***	***.72***	***.59***	.00	-.06	-.02	**.44**
4. Grammar, mean				***.61***	**.36**	-.12	.00	-.14	**.46**
5. Phonology, mean					***.60***	-.04	-.06	.00	.28
6. Processing speed, mean						.01	-.03	.21	.00
7. Vocabulary, difference							**.39**	**.42**	.06
8. Grammar, difference								**.34**	.09
9. Phonology, difference									-.19
10. Processing speed, difference									

Note. Pearson correlation coefficients with significant ($p \leq .05$) correlations in bold (and cursive when $p \leq .001$). Nonverbal mental functioning was assessed with the *Block Design* subtest. Mean and difference represent combination and difference between receptive and expressive functioning within each subdomain. Receptive and expressive functioning were assessed with *British Picture Vocabulary* and *Picture Naming* for vocabulary, the *Test for Reception of Grammar* and the *Grammatic Closure subtest from the ITPA* for grammar, the mean of four *Phonological awareness tests* and the *Children's Test of Non-Word Repetition* for phonological skills, and the *Child Language and Learning's speed of processing tests* and the *Rapid Automized Naming task* for processing speed. Data are based on 50 multiple imputed datasets for 0.7% missing data.
